# P-338. Real-World Adherence Patterns and Characteristics of Patients Initiating Injectable Cabotegravir for Pre-exposure Prophylaxis (PrEP) or HIV Treatment

**DOI:** 10.1093/ofid/ofaf695.557

**Published:** 2026-01-11

**Authors:** Yvonne Viteri, Duy Do, Stefanie Pitts, Angela Inneh, Gail Bridges

**Affiliations:** Accredo Specialty Pharmacy, Northvale, NJ; Evernorth Research Institute, Long Beach, California; Accredo Specialty Pharmacy, Northvale, NJ; Evernorth Research Institute, Long Beach, California; Accredo Specialty Pharmacy, Northvale, NJ

## Abstract

**Background:**

Effective viral control using daily oral antiretroviral therapy (ART) for HIV requires optimal adherence. Injectable cabotegravir/rilpivirine offers less frequent administration with comparable efficacy to oral ART. Injectable cabotegravir for pre-exposure prophylaxis (PrEP) requires less frequent dosing compared to oral PrEP, which may impact adherence. Understanding adherence patterns is crucial for optimizing real-world effectiveness.

**Methods:**

Adherence was assessed in treatment-naïve patients with an index pharmacy claim from January 2021 through December 2022 for long-acting HIV treatment (patients with ≥1 claim for injectable cabotegravir/rilpivirine) or PrEP (patients with ≥1 claim for injectable cabotegravir). Adherence was defined as initiation and completion of initial and follow-up doses as prescribed. Logistic regression was employed to assess the likelihood of adherence within both treatment cohorts based on certain demographic and clinical characteristics: age, sex, race/ethnicity, geographic region, Charlson comorbidity index, insurance type, and social determinant of health index. Results are presented as odds ratios (OR) with corresponding 95% confidence intervals (CI). Statistical significance was set at p< 0.01.

**Results:**

We identified 916 PrEP and 1,085 HIV treatment patients. Non-adherence was more prevalent among PrEP vs HIV treatment patients (29% vs 17.4%, p < 0.001). Within the PrEP cohort, individuals residing in the South (OR=0.46, 95% CI: 0.29-0.75, p=0.002) and Midwest (OR=0.55, 95% CI: 0.34-0.91, p=0.018) regions had significantly lower odds (54% and 45%, respectively) of adherence compared to the northeast. There were no statistically significant associations with treatment adherence and other patient characteristics examined in either HIV treatment or PrEP cohort.

**Conclusion:**

Despite reductions in administration frequency and pill burden with long-acting injectable ART, 29% of injectable PrEP patients have sub-optimal adherence. Our findings highlight the need for targeted interventions and education to support patients receiving long-acting injectable ART for both PrEP and HIV treatment, particularly focusing on regional disparities within PrEP populations.

**Disclosures:**

Stefanie Pitts, Pharm.D., Accredo: Employment|Accredo: Stocks/Bonds (Public Company) Gail Bridges, PharmD, Accredo Specialty Pharmacy: Employee

